# Peptide-based coacervates in therapeutic applications

**DOI:** 10.3389/fbioe.2022.1100365

**Published:** 2023-01-04

**Authors:** Lilusi Ma, Xiaocui Fang, Chen Wang

**Affiliations:** ^1^ CAS Key Laboratory of Biological Effects of Nanomaterials and Nanosafety, CAS Key Laboratory of Standardization and Measurement for Nanotechnology, CAS Center for Excellence in Nanoscience, National Center for Nanoscience and Technology, Beijing, China; ^2^ University of Chinese Academy of Sciences, Beijing, China

**Keywords:** peptide, coacervate, liquid liquid phase separation, self-assembly, complex assembly

## Abstract

Coacervates are droplets formed by liquid‒liquid phase separation. An increasing number of studies have reported that coacervates play an important role in living cells, such as in the generation of membraneless organelles, and peptides contribute to condensate droplet formation. Peptides with versatile functional groups and special secondary structures, including α-helices, β-sheets and intrinsically disordered regions, provide novel insights into coacervation, such as biomimetic protocells, neurodegenerative diseases, modulations of signal transmission, and drug delivery systems. In this review, we introduce different types of peptide-based coacervates and the principles of their interactions. Additionally, we summarize the thermodynamic and kinetic mechanisms of peptide-based coacervates and the associated factors, including salt, pH, and temperature, affecting the phase separation process. We illustrate recent studies on modulating the functions of peptide-based coacervates applied in biological diseases. Finally, we propose their promising broad applications and describe the challenges of peptide-based coacervates in the future.

## 1 Introduction

Liquid‒liquid phase separation (LLPS) is the foundation for the generation of membraneless organelles (Aumiller et al., 2016; Gaur et al., 2022), leading to unique regions and compartments in cells, such as the nucleolus and Cajal bodies in the nucleus and stress particles and P granules in the cytoplasm. Multivalent interactions are the main driving force for LLPS. Phase separation participates in several functional biological activities (Nakashima, et al., 2019), such as DNA damage repair (Patel et al., 2015), signal transmission (Sabari et al., 2018), gene expression and modulation of peptide assembly (Bari and Prakashchand, 2021).

With high loading efficiency *via* electronic interactions, excellent biocompatibility, membrane permeability, and low cytotoxicity (Xiao et al., 2020; Shirzadian et al., 2021), coacervates have more extraordinary advantages than traditional nanoparticles (Jo et al., 2019). Coacervates have already been confirmed to be utilized as a protein delivery system to accelerate skull bone regeneration (Kim et al., 2018), tumor treatment (Liu, et al., 2022) and neurodegenerative diseases (Wu et al., 2018). Moreover, coacervates formed by histidine-rich beak protein derivative peptides can not only deliver macromolecules into cells but also protect mRNA from degradation (Sun et al., 2022). Therefore, coacervates would be considered as delightful therapeutic agents, especially in drug delivery systems, tissue regeneration and biomimetic protocell (Matveev, 2019).

Overall, various polymers participate in the coacervation process, such as proteins, nucleic acids, and polysaccharides. In particular, intrinsically disordered regions (IDRs) are a common component driving phase separation, which provides a foundation for peptides to be potential candidates for coacervates. Peptides and their derivatives are involved in phase separation and they are easily synthesized, accessibly designed and highly produced, which would be an ideal model for experiments.

Peptides have a simple backbone chain and various functional groups in their side chains, contributing to the molecular self-assembly (Liu, et al., 2021). They can form special structures, including fibers, nanotubes, nanowires, etc. In addition, hydrophobic interactions and hydrogen bonding in peptide chains contribute to special conformations, such as α-helices and β-sheets, leading to peptide self-assembly and complex assembly (Zhou et al., 2017). Peptide assembly are closely related to many intractable diseases (Yu et al., 2017) and involved in many processes of constructing supramolecular structures and nanomaterials (Zheng et al., 2019). Due to the advantages of their structures, peptides are sensitive to external stimuli. Their structure contains different moieties that contribute to multiple intramolecular and intermolecular interactions through several conditions, leading to phase separation. Additionally, in phase separation, peptides can form coacervates by self-assemblies or complexes with other components due to their special sequences. Modulation of peptide assembly through ion strength, pH, and temperature would be conducive to forming droplets, which has attracted increasing attention to peptide-based coacervates (Sheehan, et al., 2021). Moreover, numerous studies have focused on the interactions in phase separation droplet. It is crucial to investigate intramolecular and intermolecular interactions between peptides and other peptides with different sequences, and also other molecules such as nucleic acids, and polysaccharides, which contributes to important physiological functional LLPS.

**FIGURE 1:**
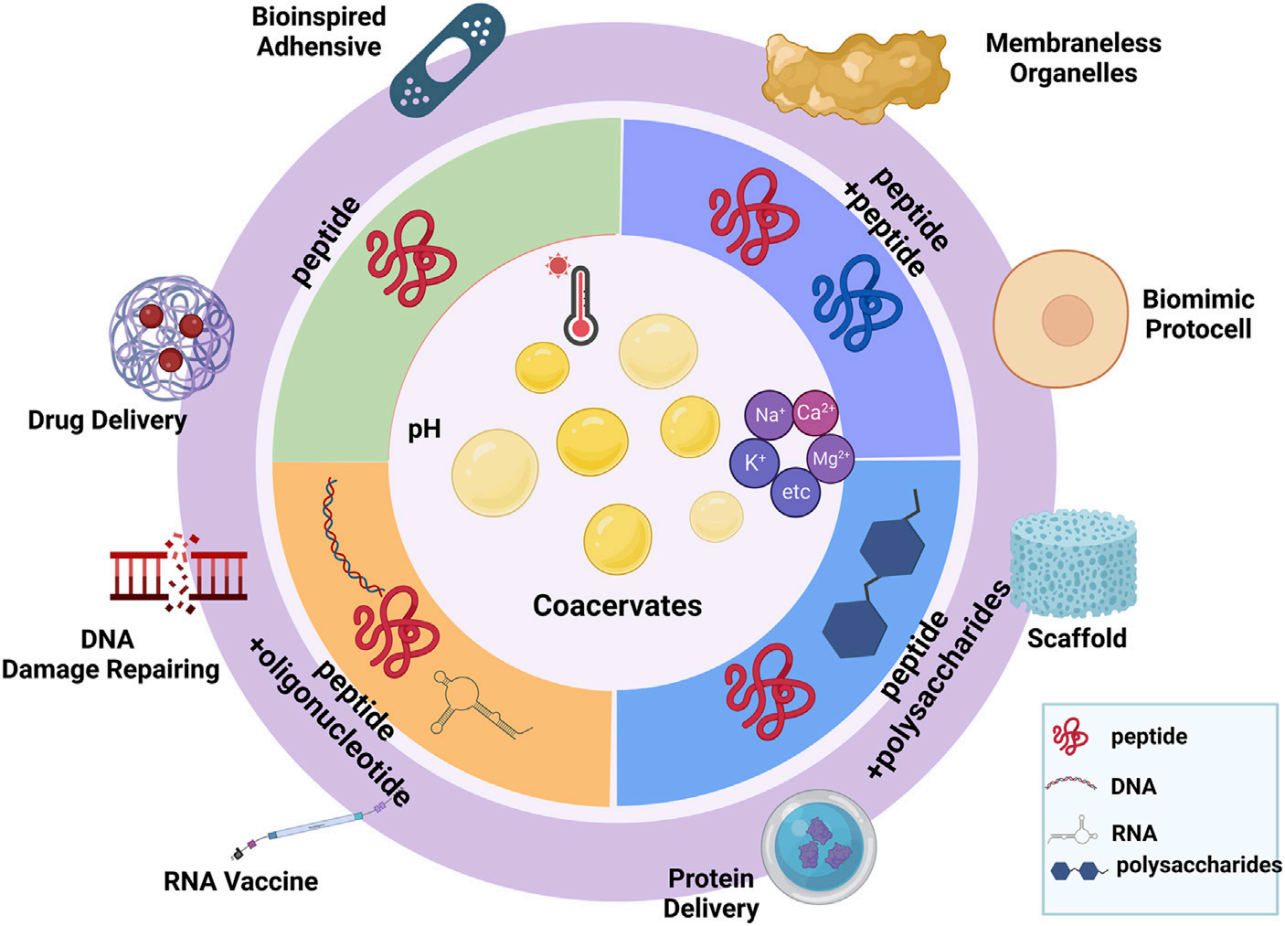
Summary of different types of peptide-based coacervates. (Created with BioRender.com, accessed on 13 November 2022).

In this review, we focused on peptide-based coacervates, which are considered to have novel therapeutic applications (Figure 1). First, we introduced the background of condensates and provided the main driving forces for the coacervates. Additionally, we explored the influencing factors that contribute to coacervation, such as ionic strength, pH, temperature, and thermodynamic principles. According to recent studies, we sorted the peptide-based coacervates into different types based on their interaction components. Mainly, we discussed the peptide-peptide interactions, peptide-DNA interactions, peptide-RNA interactions, and peptide-polysaccharide interactions leading to liquid‒liquid phase separation and coacervates formation. Finally, we demonstrated examples of therapeutics, which is probably a significant step toward the development of novel applications in therapies.

## 2 Peptide-based coacervates

Numerous reports have demonstrated that peptides are able to enter a dense liquid phase from a dispersed solution. Peptides form different structures in a dense liquid phase due to the various participants. It could be a simple coacervate formed by only one peptide but it could also be a complex coacervate that may contain peptides, nucleic acids or polysaccharides. The participants affect the properties of the coacervates as well as their applications.

### 2.1 Peptide-peptide coacervates

Peptide-peptide coacervates can be divided into two types: simple coacervates, which are composed of only monomeric peptides, and complex coacervates, which are composed of two different types of peptides.

Self-coacervate peptides are important components of underwater adhesives, drug delivery systems and even the origin of life, although they are usually not native peptides or proteins. Studying artificially designed self-coacervate peptides provides evidence of the influence of the primary amino acid sequence or composition on coacervation (Wei et al., 2016). In addition, self-coacervation could be a reasonable model for studies of the origins of life. A Summary of self-coacervate peptide is showed in Table 1.

**TABLE 1:** Summary of self-coacervate peptide.

Peptides	Sequence	Notably details	References
DgHBP-2	GHGVYGHGVYGHGPYGHGPYGHGLYW	glucose-responsive insulin delivery system; encapsulated with magnetic nanoparticles and Doxorubicin(Dox) as a liver cancer treatment	Lim et al. (2018), Lim et al. (2020)
mfp-3S-pep	GYDGYNWPYGYNGYRYGWNKGWNGY	Systemic underwater adhesive	Kaminker et al. (2017), Wei et al. (2016)
ATBP	SKGPG(AGVPG)_160_(YG)_6_(CGG)_8_WP	Delivery system for hydrophilic small molecules like water-soluble drugs and imaging agents	Bhattacharyya et al. (2017)
vRAGE-ELP	MAQNITARIGEPLVLKCKGAPKKPPQRLEWKLNTGRTEAWKVLSPQGGGPWDSVARVLPNGSLFLPAVGIQDEGIFRCQAMNRNGKETKSNYRVRVYQIPGGGGSGGGGSGGGGSHMGPGVGVPGVGVPGVGVPGVGVPGVGVPGVGVPGVGVPGVGVPGVGVPGVGVPGVGVPGVGVPGVGVPGVGVPGVGVPGVGVPGVGVPGVGVPGVGVPGVGVPGVGVPGVGVPGVGVPGVGVPGVGVPGVGVPGVGVPGVGVPGVGVPGVGVPGVGVPGVGVPGVGVPGVGVPGVGVPGVGVPGVGVPGVGVPGVGVPGVGVPGVGVPGVGVPGVGVPGCGVPGVGVPGVGVPGVGVPGVGVPGCGVPGVGVPGWPTSQPELAPEDPEDVEHHHHHH	Promising therapy for diabetic wound	Kang et al. (2021)
NTA-Fn	(FPGVG)_n_ *n* = 3 or 5	Potential strategy of capturing heat-responsive molecule and metal-scavenging agents	Suyama et al. (2021)
I-ELP	(VPGVG)_120_(GY)_7_	Application for intratumoral radiation therapy	Schaal et al. (2016)
YFsFY	YFsFY	Tyrosine rich short peptide was controlled by pH window and enable oxidative crosslinking	Abbas et al. (2022)
SUP	(VPGXG)n	Supercharged ELP derived peptide improve wound healing and skin regeneration	Sun et al. (2021)
	X=lysine (K) or glutamic acid (E)		
3KV84	MGKKKP (VPGVG)_14_ (VPGVG)_14_ (VPGVG)_14_ (VPGVG)_14_ (VPGVG)_14_ (VPGVG)_14_	Amino-acid side chains had different sensitivity to the coacervation	Singh et al. (2016)
DCH_PIC_	(M^O^A)_155_E_30_	Form a new supermolecules morphology in polyion complex	Sun et al. (2017)
	(M^O^A)_156_E_27_		
	(M^O^A)=poly(Lmethionine sulfoxide-stat-L-alanine)		
LVFFAR_9_	LVFFAR_9_ + ATP	Investigated the properties supermolecule amyloid coacervates	Jain et al. (2022)
Dendritic ELP	(GLPGL)_n_	Structure-property relationships of dendritic ELP	Navon et al. (2016)
ELP-1	GPG(VPG[V_5_G_3_A_2_]G)_150_WP	Loaded Dox to temperature-sensitive targeted delivery	Zai-Rose et al. (2018)
F_5_	H-(FPGVG)_5_-OH	Effects of aromatic amino acids on ELP	Suyama et al. (2022)
ELP_3_	AAAAAKAAKYGA [(GVPGV)_7_ AAAAAKAAKYGA]_3_	A new generation method for elastic fiber	Lau et al. (2022)
P_D_OC or P_DL_OC	poly(_D_ -ornithine-co-citrulline)	Stereoregularity influences UCST behaviors	Kuroyanagi et al. (2019)
	poly(_DL_ -ornithine-co-citrulline)		
PEG-ELP	MASMTGGQQMG-HHHHH-DDDK-LQ[LDAS-TVYAVTGRGDSPASSAA-SA((VPGIG)_2_VPGKG(VPGIG)_2_)_3_VP]_4_LE	PEG enhanced ELP’s ability of release, hydrogel storage and optical density	Meco and Lampe (2019)
ELP-CLP	(GPO)_8_GG, (VPGYG)_y_(VPGFG)_x_G	Influence of tyrosine residues in ELP	Taylor et al. (2020)
ELP-collagen	(VPGVG)_120_	A new biopolymer combined ELP with collagen to considered as collagen scaffolds	Amruthwar et al. 92013)
REP	(TGPG [VGRGD(VGVPG)_6_]_20_WPC	Enhance the insulin secreting ability	Lee et al. (2019)
ELP[M1V3-40]	MW[VPGVGVPGMG(VPGVG)_2_]_40_	As an artificial IDR model to investigate temperature-responsive organelle-mimics	Zhao et al. (2021)
ELP90	(Val-Pro-Gly-Xaa-Gly)_90_	Transmission of ELP may not relate to residue structure	Selig et al. (2018)
	Xaa=Val, Leu, and Gly in a 5:2:3 ratio		
SOP-IDP	GVGVP	New mimic model for the thermal compactions in dominantly hydrophobic IDPs	Baul et al. (2020)
SAP	AEAEAKAKAEAEAKAK	Possess fibrillization properties and an Fc-binding function as antibody delivery system	Pham et al. (2019)

One of the most common studied peptides is mussel foot protein homolog peptide, inspired by the fact that mussels secrete a complex fluid that consists of underwater adhesive proteins. The mussel foot protein homolog peptide has strong potential for applications in bioadhesive materials in liquid environments. Kaminker et al. reported the unique development of synthetic underwater adhesives (Kaminker et al., 2017). They focused on a single mussel foot protein-3S homolog peptide that displayed suitable properties for underwater adhesives due to the combined contributions of hydrophobic, hydrogen bond, van der Waals and electrostatic interactions that are toggled by the pH and temperature. The UCST behavior proved that the interactions formed the coacervate instead of increasing the entropy in this system.

Wei et al. (2016) designed mfp-3S-pep (GYDGYNWPYGYNGYRYGWNKGWNGY) that enables the formation of simple component coacervates at suitable pH values and salt concentrations. They demonstrated that a high ionic strength and a high pH value would promote the transition from a solution to coacervation. The increase in hydrophobic interactions and short-range electrostatic attraction led to the net attraction of the peptide chain. 3,4-dihydroxy-l-phenylalanine (Dopa), known as an important structure of mussel adhesive protein, was incorporated into mfp-3S-pep to increase its adhesive ability. It has been proven that Dopa enhances the hydrophilicity of peptides and intramolecular interactions due to the H-bond between Dopa and the amino residue. With Dopa functionalization and coacervation, mfp-3S-pep was allowed to adsorb on a rough surface under solution conditions, which could be considered an inspiring development for synthetic adhesives in aqueous environments.

It has been proven that under suitable conditions, a single peptide enables proliferation, self-assembly and the formation of droplets through liquid‒liquid phase separation in solution, and the droplets have a stable growth-division period *via* autocatalytic self-reproduction (Matsuo and Kurihara, 2021). They demonstrated that the droplets would continuously grow with the addition of DTT and a disulfide precursor of monomer (Mpre) (nutrition). The size of the coacervates increased from approximately 40 nm to a very large size after 24 h, which proved that the LLPS-formed coacervates aggregated with time. They also illuminated that recursive growth and division were occurring by observing the diameters of the droplets at different times. They evaluated six periods that added Mpre and then the newly formed coacervates extruded *via* a syringe. They found that from the second to sixth cycle, the size of the droplets at the beginning and the end were basically the same, while the growth process of the coacervate was not influenced. The photos from DIC microscopy showed that the recursive patterns of the changes in the diameters and the proliferation of the particles were combined with self-production and subsequent fusion. This meant than in response to the nutrition addition and extrusion, the coacervate experienced a recursive growth-division process. The coacervate formed by the monomer peptide was probably involved in the origins of life and may also be a possible candidate for the development of a self-sustainable material.

Elastin-like peptide (ELP) is another self-coacervate peptide that has attracted a lot of attention (Rodriguez-Cabello et al., 2018). ELP, a derivation of the hydrophobic domain of tropoelastin, is made of repeated units of Xaa-Pro-Gly-Xaa-Gly, where X could be any amino acid except proline (Navon et al., 2016). Due to its thermally responsive capability, it would be an ideal drug delivery system for cancer treatment (Despanie et al., 2016; Bhattacharyya et al., 2017), wound healing (Kang et al., 2021) and metal scavenging agents (Suyama et al., 2021).

To construct a highly reliable and effective therapeutic cancer treatment, an ELP-formed drug delivery system has been utilized for several different types of tumors (Schaal et al., 2016). The ELP (the peptide sequence (VPGVG)_120_ (GY)_7_) formed a thermally triggered micelle to overcome the limitations of the thermosensitive radionuclide polymer to form a potential brachytherapy delivery agent with low off-target toxicity. The coacervate-loaded β-emissions of _53_^131^ I would be triggered by body temperature to transform into an insoluble viscous coacervate, which would provide a strong and protected radionuclide. The radioactivity of _53_^131^ I remained above 52% and 70% in prostate and pancreatic tumor models, respectively, after 60 days of treatment without obvious radioactivity accrual. In a prostate mouse model, the tumor was dramatically reduced, and the volume of the tumor was less than 5% of its original volume. The same effect was reported for a pancreatic BxPc3 luc mouse model. After only 7 days, the growth of the tumors was strikingly limited, and the volume of the pancreatic tumors was less than 300 mm^3^ after the treatment was completed. Compared with the surgery group, the brachytherapy treatment group showed little body weight loss, which proved the excellent biosafety of ELP micelles. The smart thermal response mechanism suggests a wide and potential therapeutic future for ELPs.

Peptide-peptide coacervates can be formed by two different charged peptides, poly-lysine and poly-glutamate, whose electronic interactions are their main driving force (McCall et al., 2018). However, hydrogen bonding, chirality, hydrophobicity, the length of the peptide chain, the charge density and the chemical properties of the functional groups are important. A summary of peptide-peptide coacervates is exhibited in Table 2. Tabandeh and Leon, (2019) reported coacervates formed by polycations and polyanion peptides. They exchanged the peptide sequences of D- and L-chiral patterns of lysine or glutamic acid for glycine, alanine or leucine. They demonstrated that with increasing hydrophobicity of the peptides, the loading efficiency of coacervates that worked as a novel drug delivery system was significantly enhanced.

**TABLE 2:** Summary of peptide-peptide coacervates.

Peptide	Peptide	Notable details	References
poly-L-lysine	poly-(L,D)-glutamic acid	Self-assembling polypeptide coacervates to investigate the partitioning of cytoskeletal protein actin into liquid polymer rich droplets	McCall et al. (2018)
V96	humanin peptide	Humanin peptide assembled with ELP to protect human retinal pigment epithelium cells from death	Li et al. (2020)
ɣ-poly-glutamic acid	ɛ-poly-L-lysine	Oral delivery	Muriel Mundo et al. (2020)
TDP-43 CTD	GRN-5	Verify GRN participated in autophagy of TDP-43 in redox condition	Bhopatkar et al. (2020)
Poly(L-lysine hydrochloride)	poly(L-glutamic acid	Systematic research of the interfacial energy in coacervates	Priftis et al. (2012)
	sodium salt)		
Poly(g-3-(4-(guanidinomethyl)-1H-1,2,3-triazol-1-yl)propyl-l-glutamate (PPLGPG)_50_	PolyGlu_50_	The influence of α-helix in coacervates	Priftis et al. (2015)
PLys_50_			

### 2.2 Peptide-DNA coacervates

Single stranded DNA (ssDNA) and double stranded DNA (dsDNA) exhibited strikingly different behaviors in complex with a polycation peptide. Compared with dsDNA, ssDNA is a shorter, more flexible and hydrophobic polymer with a lower charge density. These factors have strong effects on the formation of coacervation (Vieregg et al., 2018). A summary of peptide-DNA coacervates is showed in Table 3.

**TABLE 3:** Summary of peptide-DNA coacervates.

Peptide sequence	DNA name	Application	References
CKKKHHHHKKKC	pEGFP-C1	Environment sensitive gene delivery system	Wang et al. (2020)
(RRLR)6-SSSGSS	21-mer single-stranded oligonucleotides of random sequence	Mechanism of regulating membranless organelle and living cells	Bai et al. (2021)
Polylysine	5’-TGAACTAACG-3’	Dynamic regulate the reactions of LLPS	Wee et al. (2021)
(KG)_15_ and (KGG)_10_	5’-TCAACATCAGTCTGATAAGCTA-3’	Sequence-specific hybridization control	Vieregg et al. (2018)
		LLPS	
poly-L-lysine	21nt ssDNA	New non-equilibrium dynamic behaviors in artificial protocells	Yin et al. (2018)

Single stranded DNA (ssDNA) has flexible chain and non-defined conformation, which promote the development of coacervates. Wee et al. reported that the coacervates formed by polylysine and arylazopyrazole (AAP)-conjugated ssDNA would contribute to improve the observation of transient and dynamic LLPS *via* photoswitch (Wee et al., 2021). The photoswitichable droplets would control the conformation of ssDNA through irradiations of different wavelengths. Notably, the scientists demonstrated that the electronic interactions between the peptide and backbone of ssDNA drove the formation of coacervates and also claimed that the cis-trans isomerization of units which were absent from binding activity contributed to the interactions in droplets. The findings illuminated that the coacervates enable to improve the acceleration in kinetics both in catalyzed and uncatalyzed activities.

Long double stranded DNA has rarely been studied in peptide-based coacervation due to its stiff chain, hydrophilicity and high charge density. However, some short dsDNA recently shifted the perspective when a coacervation containing short double-stranded DNA (dsDNA) and poly-l-lysine (PLys) was formed and contributed to the exploration of probiotic cell evolution (Fraccia and Jia, 2020). The coacervates of short dsDNA and poly-l-lysine exhibited liquid‒liquid phase separation instead of precipitation, and the short dsDNA dominated the aggregation and packing process in coacervation. Additionally, the phase behaviors were controlled by the temperature, monovalent salt concentration, dsDNA, and peptide concentration, which kept the complex in a fluid state. It also offered opportunities to generate multiphase condensates, which provided an understanding of how nucleic acids and peptides self-assemble to evolve prebiotically together.

Furthermore, hybridization of the peptide chains influenced the behavior of phase separation due to the difference in charge density (Vieregg et al., 2018). (KG)_15_ and (KGG)_10_ affected the size of the coacervates from approximately 100 nm-10μm, although the phase formed with ssDNA was not changed.

### 2.3 Peptide-RNA coacervates

Membraneless organelles were confirmed to play an important role in living cells, affecting the process of disease. Because of their capability of imbibing biomolecules, membraneless organelles could be a location of multiple reactions. Peptide-RNA coacervates are generated through phase separation, with multivalent associations (Saha et al., 2019). RNA, as an anionic polyelectrolyte, has a highly negatively charged phosphate backbone. Electronic interactions are one of the main forces of coacervation; RNA is able to combine with cation polypeptides to form droplets, and the condensate is involved in various physiological activities (Henninger et al., 2021). A summary of peptide-RNA coacervates is showed in Table 4.

**TABLE 4:** Summary of peptide-RNA coacervates.

Peptide	RNA	Notable details	References
polyamines	polyU	Simplified model to explore the property of interfacial liposome assemble for membraneless organelles	Aumiller et al. (2016)
Primordial-(HhH)2-Arg	polyU	Disordered polypeptides can give rise to structure	Longo et al. (2020)
Primordial-(HhH)2-Orn			
K_4_, K_10_	polyU	New explanations for temperature-dependent drive force	Pullara et al. (2022)
R_10_, K_10_	Template-directed RNA	Enhance the functions of RNA and other biopolymers	Poudyal et al. (2019)
K_72_	(ACGU)6	High catalytic efficiency	Nakashima et al. (2021)
(PR)_12_	PolyA	Poly(PR) inhibited the process of transcription and diffusion of NPM1	Chen et al. (2021)
TDP-43 CTD	torula yeast	Leading to TDP-43 pathobiology	Bhopatkar et al., (2020)
	RNA		
R_10_	RNA 15-mer	Explore the mechanism that short non-code peptide enhance the early cell fitness	Jia et al. (2016)
R_4_	rIGSRNA	Long and low complexity RNA is important to build membrane-less compartments	Wang et al. (2018)

The structure of the peptide determines the formation of coacervation, which contributes to the protein structure domain (Seal et al., 2022). Recently, a flexible peptide and its derivatives showed that a helix-hairpin-helix (HhH) motif combined with RNA contributes to coacervation. The flexible peptide was present as dimers alone, which built bridging bidentate interactions with PolyU in coacervates. The results from double electron−electron resonance spectroscopy showed the symmetric (HhH)_2_-fold that was related to dsDNA binding in the distance distribution between dimers and α-helix folding. Based on this study, they claimed that peptide aggregation was the special aspect of coacervation and peptide-involved liquid‒liquid phase separation would be the basis of the formation of protein structures in duplication and fusion processes in original life. Aumiller et al. proved that shifting peptide phosphorylation would control peptide-RNA droplet coacervation (Aumiller and Keating, 2016). Adding or removing a phosphate site from the peptide (peptide sequence: RRASLRRASL) led to the aggregation or disaggregation of the droplets because the electronic interactions in the coacervates were changed. Similar results were found for simple base RNA (poly U) and transfer RNA, which contain four bases and have a molecular weight of approximately 23–27 kDa.

In addition, peptide-RNA coacervates contribute to enhancing the catalytic ability of RNA (Poudyal et al., 2019). Le Vay et al. recently reported that in coacervation formed by poly-l-lysine (PLys) and a split HPz ribozyme, the ribozyme activity was increased significantly in different circumstances. The variant chain length of PLys showed that regardless of it being a longer peptide (PLys)_19–72_ or a shorter peptide (PLys)_5–24_, a polycation peptide improved ribozyme activity (Le Vay et al., 2021). This ability was suppressed at a high ratio of peptide, while at a low ratio, (PLys)_19–72_: RNA = 1.1:1 and (PLys)_5–24_: RNA = 2.3:1, the catalytic ability was strong. The polycation peptide enhanced ribozyme activity through electronic-mediated liquid‒liquid phase separations, leading to peptide-RNA coacervation instead of cleavage. Therefore, short motifs offer the chance for RNA to form long and complex chains without external simulation, which supports the hypothesis that the peptides and RNA were undergoing early coevolution. Additionally, simple heteropeptide-RNA coacervates have been demonstrated to enable the maintenance of ribozyme activity (Iglesias-Artola et al., 2022).

The concentration of Mg^2+^ is one of the standards used to evaluate the level of ribozyme activity because Mg^2+^ is a necessary factor for the catalysis of RNA. Although the results of ICP‒MS and ITC compartment peptide (RGG)_4_ coacervation had higher mobility than R_9_, K_9_ and Mg^2+^ partitioning was dependent on the change density of the peptide. (RGG)_4_, which had a lower change density, had a lower binding ability to RNA. The heteropeptide had a larger transition area than the homopeptide, leading to a wider range of peptide concentrations to guard the catalytic ability of the RNA. Therefore, the properties of the peptide sequence would dominate the activity of the ribonucleic acid, and coacervation between the peptide and RNA could force the development of protocellular compartments.

### 2.4 Peptide-polysaccharides coacervates

Peptide-polysaccharides coacervation has been widely used in food science (Glab and Boratynski, 2017; Niu et al., 2018), drug delivery system (Abashzadeh et al., 2011; Park et al., 2021) and skin regeneration (Park et al., 2019). Electronics interactions drive the formation of droplet to control the structure under suitable circumstance (Pillai et al., 2021). Also, the interactions were influenced by the eternal factors such as pH, temperature and ion strength. A summary of peptide-polysaccharides coacervates in Table 5. It was proven that the coacervates contained poly-arginine or poly-lysine with heparin had high loading efficiency (Chu et al., 2012; Johnson et al., 2014). Polycation peptides combined with heparin would be an intelligent delivery strategy for growth factors. With the high loading efficiency approximately 99%, the coacervates loaded fibroblast growth factor-2 (FGF2) (Chu et al., 2011). The results demonstrated the coacervates not only offered the effective protection for FGF2 from proteolytic degradations, but also enhanced the functions of differentiation and chemotaxis with relative low dose. Chitosan, as the biocompatible cation polysaccharide, combined with anionic polypeptide poly (γ-glutamic acid) to form the condensate to deliver IFN-γ to inhibit the invasion of colorectal cancer cells (Castro et al., 2019). The dense condensate vehicle was non-toxic and biodegradable with high loading efficacy, showing narrow size distribution with diameter approximately 180 nm, excellent modulation for IL-10-stimulated macrophages and strong ability to profiler T cells. The delight peptide-polysaccharides coacervates claimed they were promising vehicles for clinical immunomodulatory therapy.

**TABLE 5:** Summary of peptide- polysaccharides coacervates.

Peptide	Polysaccharides	Notable details	References
poly(L-argininate glyceryl succinate)	Heparin	Biocompatible delivery system	Chu et al. (2012)
poly(L-argininate glyceryl succinate)	Heparin	IL-12 coacervates to treat B16-F10 melanoma	Hwang et al. (2020)
poly(L-argininate glyceryl succinate)	Heparin	A new therapy of attenuating the disc degeneration	Zhu et al. (2019)
P1 GWVDHFADGYDEVIA	Chitosan	Verify electronic interaction is main force for peptide/chitosan coacervates	Zubareva et al. (2013)
P2 MELPSFGVSGVNESADM			
P3 DQGHDTGSASAPAST			
P4 VGIDQPPFGIFV			
P5 SNCHTHEGGQLHCT			
P6 CREGEDNSKRN			
poly(γ-glutamic acid)	Chitosan	As an immunostimulatory carrier loading IFN-γ for colorectal cancer	Castro et al. (2019)
CWGGKAKAKAKAKAKAKA	glycosaminoglycan	Layer coating for ECM mimic system	Wieduwild et al. (2018)
polylysine	carboxymethyl-dextran	Offer a potential model for biomimetic protocell	Dora Tang et al. (2015)
poly(ethylene lysinylaspartate diglyceride	Heparin	Encapsulated protein as a safe and efficient delivery system	Johnson et al. (2014)
ELP	hyaluronic acid	Potential hydrophobic delivery system	Tang et al. (2018)

## 3 Mechanism of coacervation

### 3.1 Thermodynamic principles

Entropic would vary in the coacervation steps, which influences the thermodynamic changes of droplets and the inner forces of their formation. The thermodynamic process of coacervation is still a controversial topic (Singh and Yethiraj, 2020). Garcia-Rojas et al. claimed that coacervates containing ovalbumin and lysozyme had two steps of formation, including enthalpically favorable and entropically unfavorable contributions (Santos et al., 2018). The isothermal titration calorimetry data demonstrated that electronic interactions and hydrogen bonds both contributed to the interaction. Yethiraj et al. tried to explore the thermodynamics of oligomer coacervation *via* MD simulations and umbrella sampling and demonstrated that π-cation interactions played a significant role in coacervation thermodynamics (Singh and Yethiraj, 2021). Containing poly-arginine (pR) and poly-tyrosine (pY), the oligomer coacervation was influenced by its free energy, which was different from the complexation of polyelectrolytes. The coacervates had a similar free energy profile to pR and pY, indicating that oligomer complexation dominated the phase separation. Additionally, the number of π-cation interactions was related to the free energy, and it was proven that the addition of salt would contribute to hindering the π-cation interactions between R and Y or competing with R carbocation to form π-cation.

The coacervates formed by poly-l-lysine (PLys) and different types of nucleoside triphosphates (NTPs), that is, A/G/C/T/U, exhibited impressive heat-response behavior due to base stacking interactions (Lu et al., 2021). As the temperature increased, the critical salt concentrations of ATP/PLys, GTP/PLys, CTP/PLys, TTP/PLys and UTP/PLys decreased. GTP/PLys coacervates decreased strikingly compared with the other NTPs at the same change in temperature. Additionally, it was observed that as the temperature decreased, the critical salt concentrations of GTP and ATP increased faster than those of NTPs. The reason why NTP/PLys coacervates respond to temperature differently is the difference in their relative stacking free energies of their nucleobases, which has been proven by the line of the NTP changing with temperature. They also proved their hypothesis with simplified Glory-Huggins equations, and their data analysis demonstrated that the entropy and enthalpy of PLys/GTP coacervates were due to the formation of hydrogen bonds between the polycations and GTP. Wang et al. investigated the binding affinity between the nucleotides and the homo-octapeptides of 20 common amino acids and an affinity matrix of 20 common amino acids, which exhibited proof of thermodynamics between amino acids and nucleotides or between amino acids and amino acids (Du et al., 2019; Wang et al., 2022). They claimed that the interactions were influenced by the DNA backbones, DNA base types, peptide chains and side function groups, which offers wise suggestions for phase separation. In addition, Lim et al. discovered that His and Tyr residues in the termini would enable forced coacervation and become the sites of interactions (Lim et al., 2021).

### 3.2 Kinetics

**FIGURE 2:**
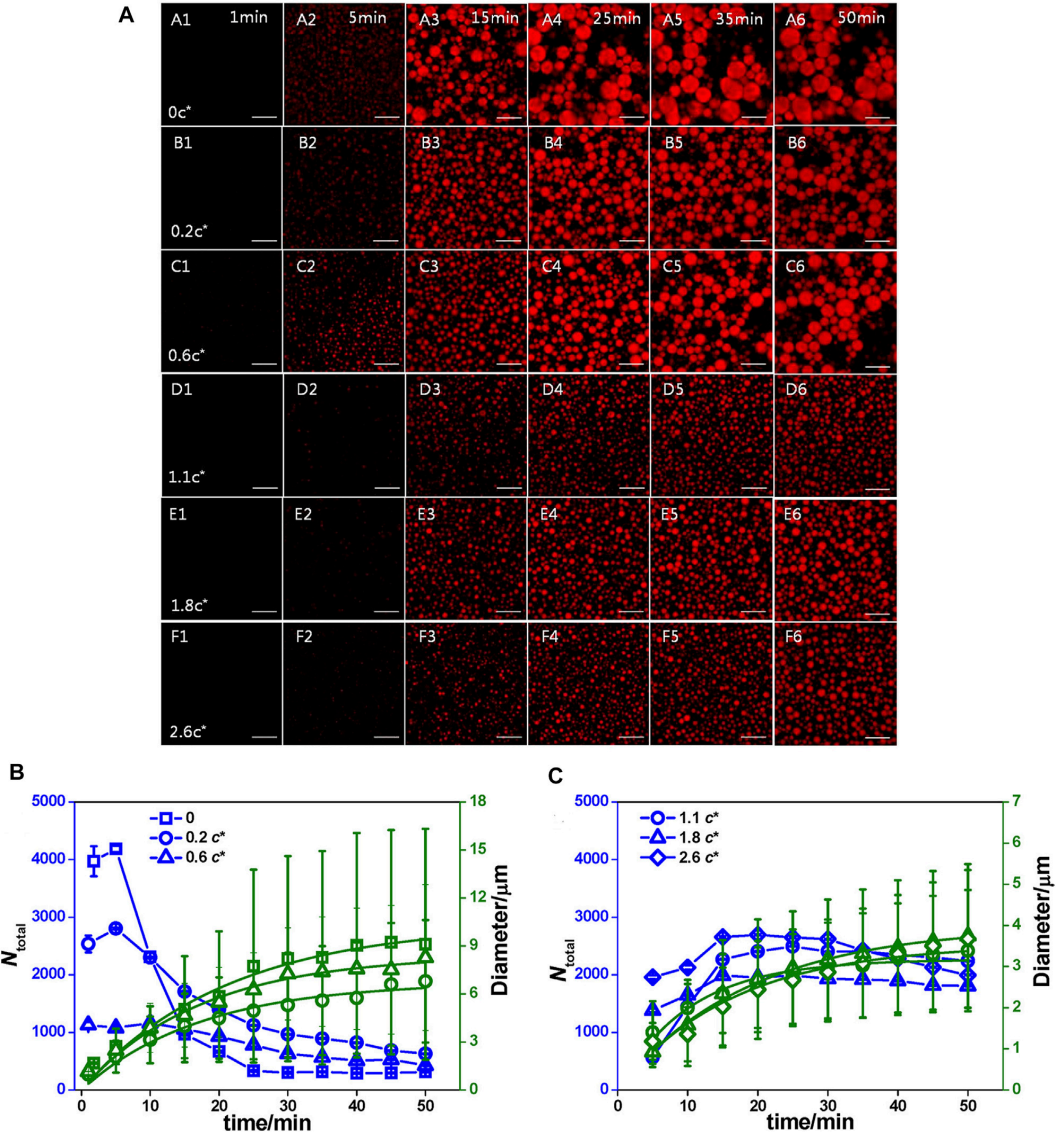
The influence of kinetics. **(A)** Time dependence of the coacervate droplets formed by 1.0 mg/mL S5 and 1.4 mg/mL ss-oligo in PAM solutions at varying concentrations as denoted by c/c*. c* is the overlap concentrations and c* of PAM is 2.7 mg/ml 1× DPBS buffer, Scale bar: 25 μm. **(B)** 1× DPBS buffer and PAM solution at concentrations lower than c* and **(C)** PAM solution at concentrations higher than c*. The blue lines are used to guide the eyes, while the green lines are the fitting curves by equation ⟨R⟩= (Kt)Q13 n (Bai et al., 2021). Copyright © 2020 American Chemical Society.

So far, the kinetics of coacervation have not been clarified. However, there are several possible explanations for the kinetics of the formation of coacervates. Bai et al. demonstrated that the dynamic process could be described by classic nucleation theory (Bai et al., 2021). They revealed that the coacervates formed by peptide S5 and oligonucleotide underwent a time-dependent process of heterogeneous nucleation, diffusion-limited growth, and Brownian motion coalescence. The changes in size with time indicated that polycation peptide S5 bound with polyanion oligonucleotide to form neutral nuclei. Because the charge of the oligonucleotide was higher than that of peptide S5, the oligonucleotide dominated the heterogeneous nucleation. Additionally, due to the different net charges on their surface, the coacervates would increase sharply and coarsen quickly (Figure 2).

In addition, the length of the peptide and the structure undoubtedly played an important role in kinetics (Spath et al., 2021). Through several cationic peptides, it was clear that with chain length growth, the level of crosslinking of coacervates was enhanced, which reduced the mobility of the droplets. Moreover, the addition of block polymer easily influenced the coacervate morphology. Scientists have tried to add poly (styrenesulfonate) to peptides as repelling segments and poly (ethylene glycol) as affinity segments to active peptides. The existence of a block polymer contributes to phase separation, while with the loss of the driving force of poly (styrenesulfonate), the dense packing, and crowding in the corona are obviously reduced, leading to breakage of the polymer**.**

## 4 Influencing factors

### 4.1 Ionic strength

Ionic strength is a significant factor that contributes to peptide-based coacervates, which usually shows variations in response to the salt concentration in solutions. Alterations of the salt concentrations would affect the formation of coacervates in two aspects: i) increasing the dissolution of components, which leads to the aggregation of coacervates, and ii) screening the net charge of the peptide and other components and hindering the interactions inside the coacervates by combining with the charged groups of the peptides or participants, which inhibit the formation of coacervates. Under a low concentration condition, the amount of coacervation increases as a consequence of the growth of dissolution. A high salt concentration usually hinders electronic interactions occurring in coacervates, leading to the suppression of coacervation.

**FIGURE 3:**
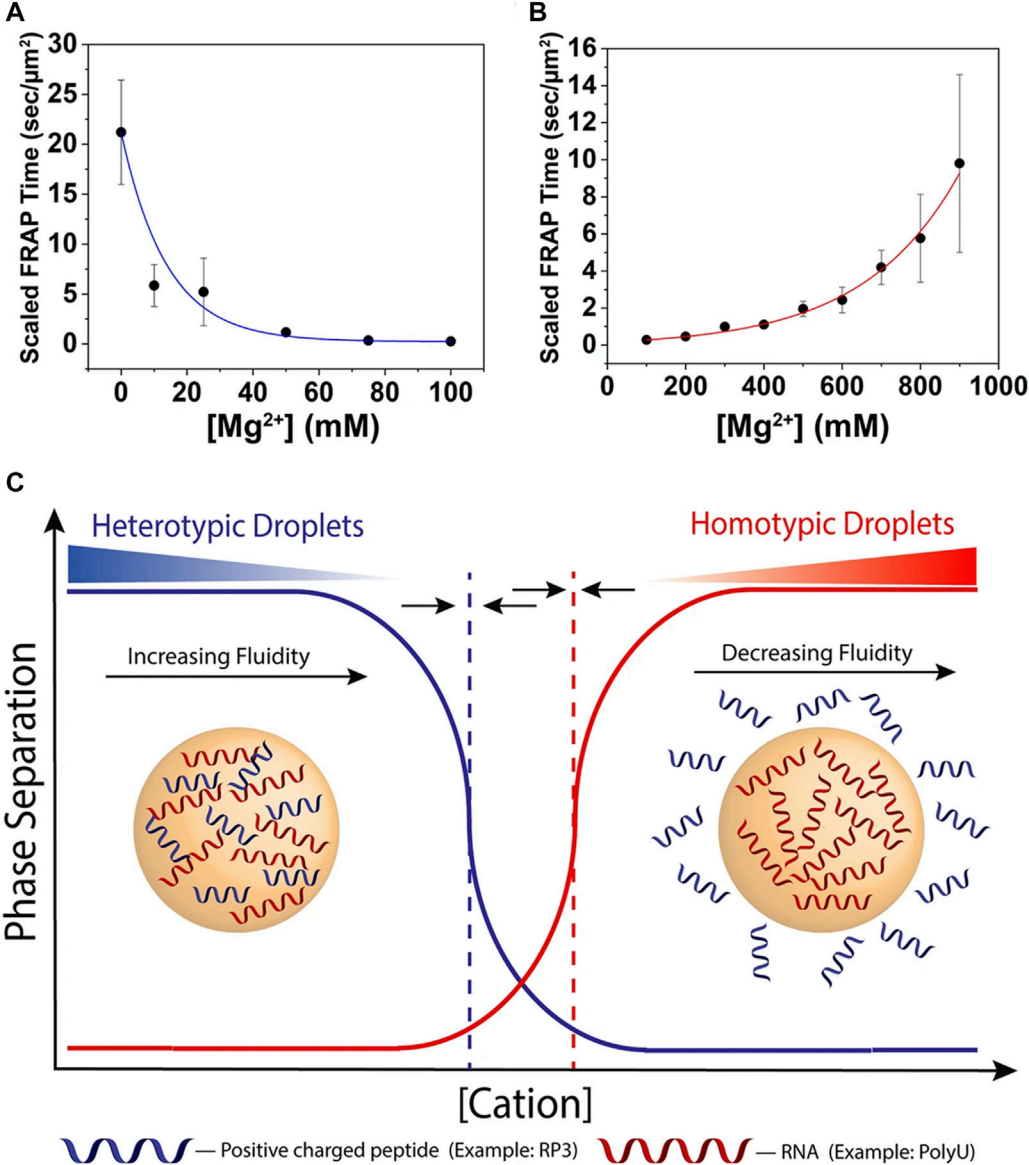
The influence of ion strength. **(A)** Scaled FRAP in heterotypic RP_3_-polyU droplets as a function of [MgCl_2_]. ([RP_3_] = 500 μM, 0.6x polyU wt/wt) The blue line is a visual guide. **(B)** Scaled FRAP in homotypic polyU droplets as a function of [MgCl_2_]. (2 mg/ml polyU, 10% PEG) The red line is a visual guide. **(C)** Cartoon representation of switch-like behavior between heterotypic and homotypic droplets as a function of increasing cation concentration. The dotted lines represent the phase boundaries of the distinct droplet types. The arrows indicate that these phase boundaries will move under changing conditions and are capable of overlapping (Onuchic et al., 2019). Copyright^©^ 2019 Springer Nature.

It has been proven that peptide-RNA droplets can be controlled by divalent ion concentrations only (Onuchic et al., 2019). Scientists took an arginine-rich peptide (RRASL)_3_ containing IDPs characteristics and polyU as an *in vitro* model. With an increasing Mg^2+^ concentration, the coacervates deceased because the electronic internationalization between the peptide and RNA was reduced, which was similar to the response to Na^+^ (Figure 3). Additionally, they explored the phase behavior with the participation of Ca^2+^ and Sr^2+^, and the concentration thresholds were 50 mM and 75 mM, respectively, while Mg^2+^ was 300 mM. The difference in the liquid‒liquid phase separation threshold was caused by their higher screening efficiency and lower capability of interactions with RNA. Altering the divalent ion concentration would be a possible solution to control the phase separation by regulating the weak interactions.

Tabandeh et al. (Tabandeh and Leon, 2019) reported two oppositely charged peptides: one with equal residues of charged and uncharged amino acids and the other with increased charge density. They explored the critical salt concentration (CSC) of the coacervates formed by the two peptides, which is an important measurement for coacervate stability. The results showed that as the salt concentration increased from 0 mM to 120 nM, the turbidity of every pair of coacervates decreased. Ion pairing with the ions in the salt solution led to screening of the opposite charges of the peptides, resulting in weakening of the electronic interactions in the coacervates and limiting their formation.

### 4.2 pH

Peptides are commonly utilized as polyelectrolytes to form coacervation *via* electronic interactions; therefore, the pH value is significant in coacervation. Usually, phase separation starts when the pH of an aqueous solution is higher than the pI of the peptides (Weinbreck et al., 2003). Mountain et al. demonstrated that in a condensate formed by gum arabic (GA) and polypeptide leucine (PL), pH affected the behavior of the polysaccharides (Mountain and Keating, 2020). The charge of the polymer chain was enhanced while the pH was lower than the pK_a_ of GA, which reduced the electronic interactions and contributed to the formation of soluble droplets.

**FIGURE 4:**
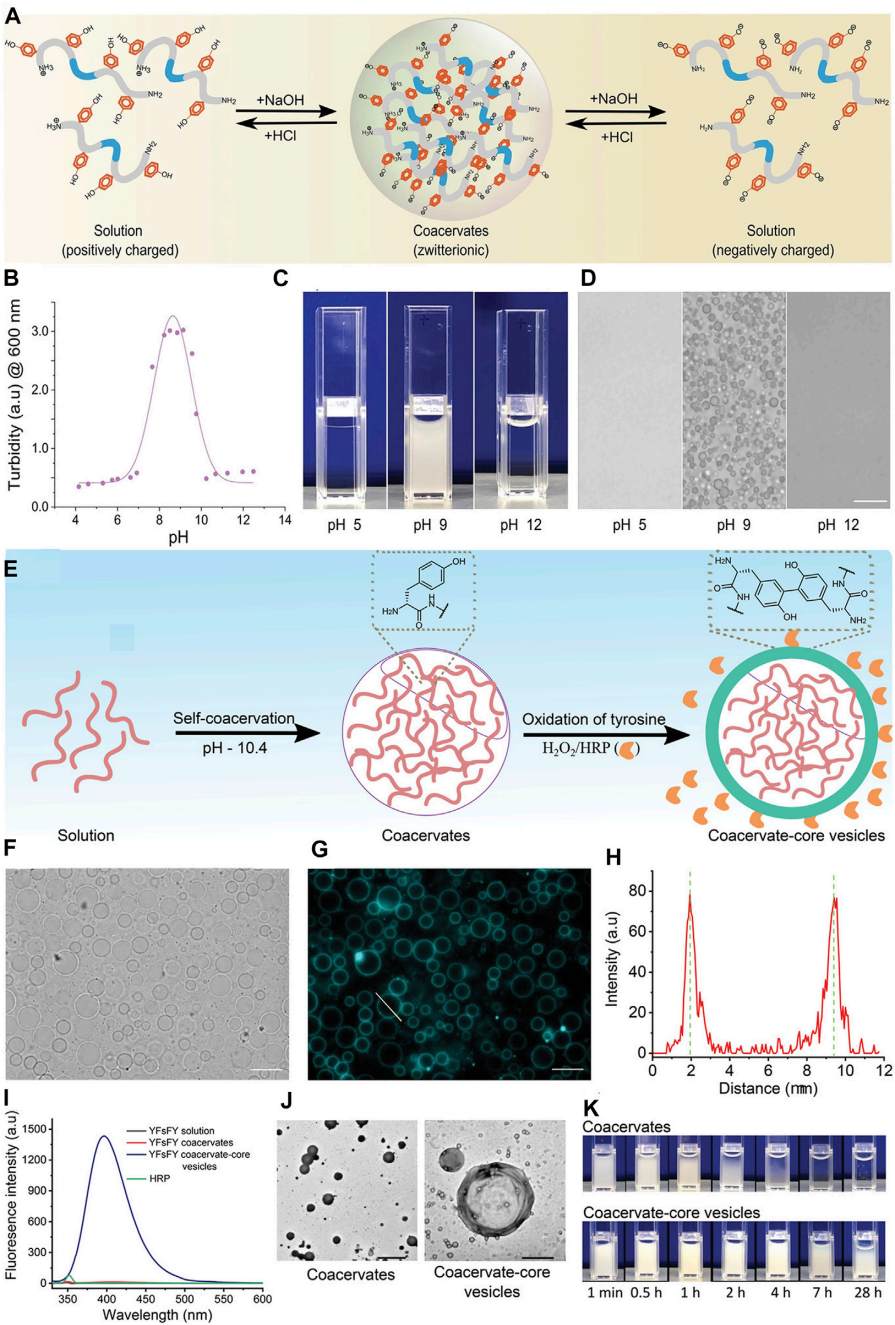
The influence of pH. **(A)** Schematic illustration of YFsFY in solution at pH 4 (positively charged), in coacervates at pH 9 (zwitterionic form) and in solution at pH 12 (negatively charged). **(B)** Turbidity of 1 mg ml^−1^ YFsFY as a function of pH with constant salt concentration of 65 × 10^–3^ M shows the formation of coacervates in a pH window between 7 and 10.5. The solid line is a fit of the turbidity data to a 2-pK_a_ model. **(C)** Appearance of 2 mg ml^−1^ YFsFY solution at pH 5, 9, and 12. **(D)** Bright-field optical microscopy of the samples in **(C)** shows the presence of coacervates at pH 9. The scale bar in **(D)** is 10 µm. **(E)** Schematic illustration of formation of CCVs. **(F)** Bright-field and **(G)** fluorescence microscopy of the CCVs formed after the oxidation of YFsFY coacervates (10 mg ml^−1^). Scale bars indicate 10 µm. **(H)** Intensity profile across a CCVs in **(G)**. **(I)** Fluorescence spectra of YFsFY coacervates before and after oxidation along with controls. **(J)** Transmission electron microscopy images of coacervates (left) and CCVs (right); scale bars: 5 and 1 μm, respectively. **(K)** Stability of CCVs in comparison with the coacervates without oxidizing agents (Abbas et al., 2022). Copyright ^©^ 2022 John Wiley & Sons, Inc.

Recently, tyrosine-rich short peptides were claimed to enable the formation of semipermeable coacervate protocells due to the oxidation of spacers in the YFsFY peptide (Abbas et al., 2022)**.** Since the phenolic side group of tyrosine had a pK_a_ of approximately 10.5, the self-assembled condensate was dissolved when the pH value increased. A condensate was observed in a range of pH values between 6.8–10.5 (Figure 4). Although the reason for this was not confirmed, it may be caused by deprotonating the cation or protonating the anion, considering that electronic interactions dominated the phase separation.

### 4.3 Temperature

Temperature is another important factor for the formation of peptide-based coacervation. The formation of hydrogen bonds and hydrophobic interactions is influenced by temperature variation (Wang et al., 2015), which contributes to polymer condensation. Recently, Li et al. demonstrated that two similar ELP sequences, poly (VGPVG)_18_ and poly (VPGVG)_18_, had different temperature-dependence behaviors in coacervates due to the decline of intrachain hydrogen bonds and the boost of interchain hydrogen bonds (Li et al., 2021). From the all-atom molecular dynamics simulation, the calculated data indicated that the different sequence orders of the peptides would lead to different structural properties. Above the transition temperature, poly (VGPVG) was more hydrophilic than poly (VPGVG) due to the wider configuration and larger surface area of the condensates. Notably, hydrogen bonds controlled the stability of the coacervation and were responsible for the chances of thermal hysteresis of the two peptides. Singh et al. observed the coacervation process through time-dependent ^1^ H high-resolution magic angle sample spinning (HRMAS) and high-resolution magic angle sample spinning (NMR) spectroscopy and found that the hydrophilic amino acid glycine α-CH_2_ was more sensitive than proline β-CH_2_ to coacervation (Singh et al., 2016).

Additionally, the particle size would be affected by temperature because ELP is thermally sensitive and is capable of coacervating reversibly with temperature shifting. Kang et al. (2021) reported that a vRAGE-conjugated ELP (peptide sequence: PLVLKCKGAPKKPPQRLEWKLNTGRTEAWKVLSPQGGGPWDSVARVLPNGSLFLPAVGIQDEGIFRCQAMNRNGKETKSNYRVRVYQIP) started to self-assemble and coacervate at approximately 31°C, and the particle had a narrower size distribution with increasing temperature. The tight size distribution would reduce any possible binding sites and enhance the protease-resistance ability.

## 5 Applications of peptide-based coacervates

### 5.1 DNA delivery system

Cell-penetrating peptides (CPPs), with excellent intracellular transition ability, are frequently considered a potential therapeutic treatment for drug delivery systems. A heptamer derivation from *Penetrain* (KIWFQNR) was reported to form coacervates with a DNA core, and the coacervation had a typical β-sheet structure (Mello et al., 2020). The peptide-DNA complex was claimed to promote cellular surface binding. The peptide complex encapsulated 200 bp DNA into HeLa cells, which was 10 times larger than other similar regulators, such as mRNA and siRNA. In addition, KIWFQNR had the longest non-charged fragment of *Penetrain*, which offers the possibility of intracellular DNA delivery without the participation of strong cations.

Wang et al. designed a bio-inspired DNA delivery system with peptide-DNA coacervate core and the dextran shield (Wang et al., 2020). They designed the positive charged peptide with sequence CKKKHHHHKKKC, while lysine enables to bind with DNA and histidine offer the proton sponge effect to release DNA. Cysteine contributed to crosslink in oxidation status in the air, which promote the coacervation. The *in vitro* results claimed that the coacervates delivery system had high DNA loading capability, high level transfection efficiency with a low cytotoxicity, which would a promising gene carrier system.

### 5.2 mRNA-based vaccines

LLPS droplets can be triggered by pH and redox responsiveness, making them suitable carriers for mRNA-based vaccines due to their excellent penetrability and biocompatibility. Sun et al. (2022) claimed to have formed microdroplets that from conjugated peptides (HBpep) and the mRNA would encapsulate EGFR-encoding mRNA into the cytosol and release it *via* the glutathione-mediated pathway. The transfection efficiency of the coacervates was higher than that of polyethylenimine and Lipofectamine 3,000 but less than that of Lipofectamine 2000 in HepG2 cells, while in the HEK293 cell line, the peptide-based coacervates had a transfection efficiency similar to that of Lipofectamine 2000. Notably, the coacervation of HBpep was friendly to cells and barely found to be cytotoxic at the optimized concentration. Additionally, they investigated the stability of the coacervates under high concentrations of RNase, and the electrophoresis experiment results showed that the molecular weight of mRNA in the condensate was barely changed. Surprisingly, compared with free mRNA, the peptide-RNA coacervates had excellent protease resistance capability, while free mRNA was rarely found after culture in RNase solution for 2 h. In summary, peptide-RNA coacervates represent a promising therapeutic platform for mRNA vaccines.

**FIGURE 5:**
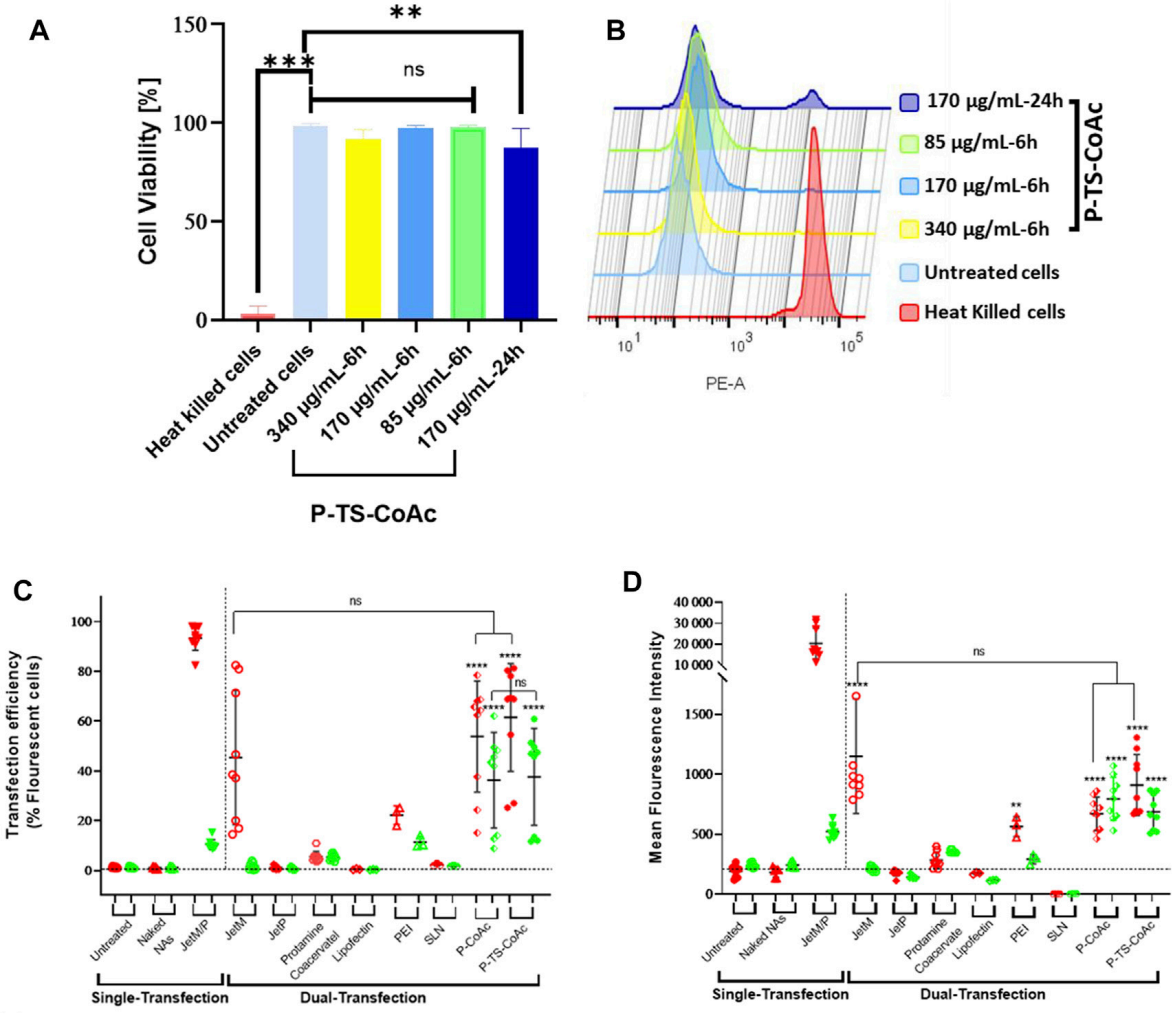
Cytotoxicity assay of P-TS-CoAc in DC2.4 murine dendritic cell line using fixable dead stain (568/583) **(A)** % Cell viability following 6 h incubation of P-TS-CoAc (340,170 or 85 μg/ml) or 24 h incubation of P-TS-CoAc (170 μg/ml) (N = 3, n = 3); **p < 0.01, ***p < 0.001, ns = not significant. **(B)** Fluorescence intensity (dead stain uptake) of cells following different treatments. Flow cytometric assessment of **(C)** transfection efficiency and **(D)** mean fluorescence intensity (MFI) of pAmCyan and mCherry loaded on P-CoAc and P-TS-CoAc, compared to single transfection using either JetMessenger for mRNA or JetPrime for DNA or double transfection using both mRNA and DNA with either JetMessenger, JetPrime, protamine sulfate (*N* = 3, *n* = 3), Lipofectin, PEI or SLN (*N* = 1, *n* = 3) coacervate in murine dendritic cell line DC2.4; **p < 0.01, ****p < 0.0001, ns = not significant. (Nasr et al., 2021). Copyright ^©^ 2021 by the authors. Licensee MDPI, Basel, Switzerland.

Nasr et al. reported coacervate nanosystem for co-delivery of mRNA and plasmid DNA (Nasr et al., 2021). The cationic peptide protamine sulfate loaded mRNA and encapsulated plasmid DNA. The flow cytometry and confocal laser scan microscopy data showed the co-delivery system have dual high transfection efficiency in DC2.4 cell line with low cytotoxicity (Figure 5). In summary, peptide-RNA coacervates represent a promising therapeutic platform for mRNA vaccines.

### 5.3 Small molecule delivery system

In recent decades, coacervate delivery systems have mostly been utilized in the food science area and are now frequently reported in therapeutic fields, such as wound healing (Lau et al., 2018), inflammation, cardiovascular diseases and cancer treatment (Schaal et al., 2016). Lim et al. developed a coacervate drug delivery system containing DgHBP-2 peptide, Doxorubicin (Dox) and magnetic nanoparticles (MNPs) as a novel liver cancer treatment (Lim et al., 2020). The DgHBP-2 peptide, derived from penta-repeats, was assembled into coacervates in phosphate buffer (pH = 9.5) while Dox and MNPs were added to the solution. The coacervates demonstrated that they were internalized by HepG2 cells without the participation of adenosine triphosphate (ATP). Additionally, the confocal data claimed that the droplets could be controlled by a magnetic field that induced a 45°C temperature, leading to the release of Dox and the dissolution of coacervation. A combination of chemotherapy and thermotherapy would enhance the effect of treatment and could be a novel therapeutic strategy for liver cancer. In addition, due to the strong electronic interactions in the coacervates, they can be used as charged molecules.

### 5.4 Biomimetic protocell

Peptide-based coacervates are promising particles for creating life-like biomimetic protocells (Nakashima et al., 2021). Because peptides have a simple chemical structure and various functional groups of side chains, the coacervates isolate and concentrate many molecules without limitations from the membrane. As a dramatic model of protocells, coacervates formed by different charged polyelectrolytes have been reported to increase enzymatic ability. Yin et al. (2018) claimed that under the situation of a direct current electric field, the coacervates showed repeated periods of vacuolization, frequency, and intensity. Strikingly, fluorescence microscopy results demonstrated that the coacervates had life-like behaviors, such as changing in size and shape, chaotic growth and fusion. In addition, an electric field influenced the kinetics of the system, which contributed to the mass transport and then tuned the location and movement of the enzyme activity to achieve the chemical activity of the protocells. Moreover, peptide-based coacervates would enable a change in the reaction rate. Compared with coacervates and microdroplets from electrospray ionization, this result indicated that with the contribution from the crowding effect, the rate of transcription was increased sharply (Stroberg and Schnell, 2018).

### 5.5 Transcriptional regulation

**FIGURE 6:**
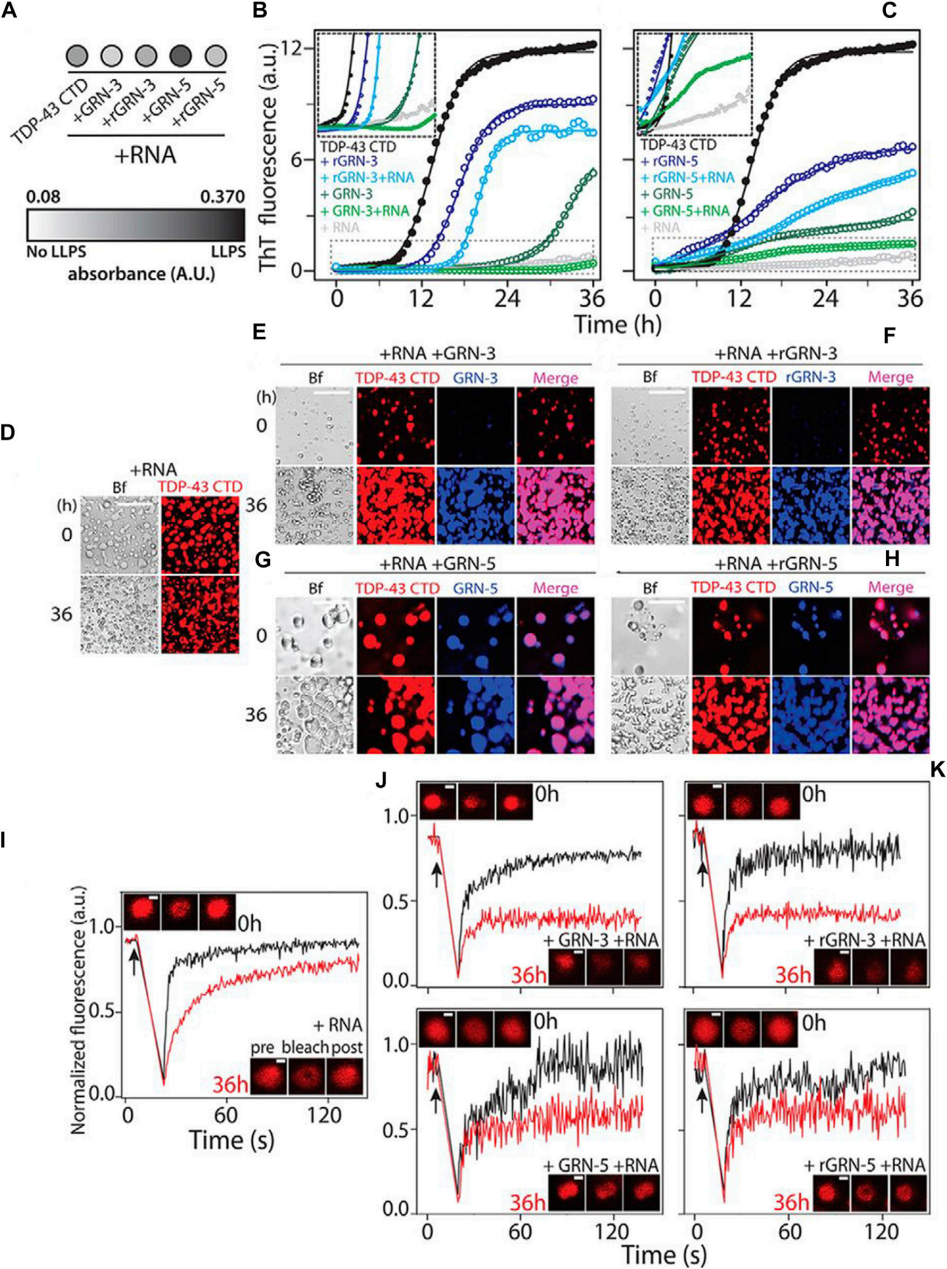
Modulation of TDP-43 CTD–RNA LDs by GRNs. **(A)** Turbidity measured at 600 nm within 10 min of incubation for co-incubated solutions of 20 μm TDP-43 CTD with 40 μm GRN-3 or GRN-5 (reduced and oxidized) in the presence or absence of 40 μg/ml RNA (*n* = 3). **(B)** and **(C)**, aggregation kinetics of 20 μm TDP-43 CTD in 20 mm MES buffer (pH 6.0) at 37°C with 40 μm GRN-3 **(B)** and GRN-5 **(C)** in both oxidized and reduced forms and in the presence or absence of RNA (40 μg/ml), monitored by ThT fluorescence. The inset shows the enlarged areas (boxed with dashed lines), highlighting the lag times during aggregation. **(D)**–**(H)**, localization of the protein from reactions similar to those in **(B)** and **(C)**, visualized by labeling the TDP-43 CTD and GRNs with HiLyte 647 and HiLyte 405, respectively, and in the presence and absence of unlabeled RNA by DIC microscopy. A control sample containing the TDP-43 CTD and RNA shows LDs **(D)**. GRN-3 **(E)** and rGRN-3 **(F)** do not localize within LDs but form solid inclusions, but GRN-5 **(G)** or rGRN-5 **(H)** colocalize within LDs. Scale bars = 20 μm **(D–H)**. **(I–M)**, FRAP data using the HiLyte 647–labeled TDP-43 CTD with LDs and GRNs immediately after incubation (0 h, black) and after 36 h (red). Arrows represent the time of bleaching. Shown are fluorescence recovery for samples containing control LDs **(I)**, GRN-3 **(J)**, rGRN-3 **(K)**, GRN-5 **(L)**, or rGRN-5 **(M)**. The insets show confocal images of the area bleached before, after, and after recovery from left to right. Scale bars = 2 μm. (Sabari et al., 2018), Copyright ^©^ 2022 Elsevier **(B)** V.

Transcription factors and cofactors, important components of transcription, have intrinsically disordered low-complexity domains leading to the formation of coacervates. Peptide-based coacervates offer the possibility of compartmentalizing and concentrating biochemical reactions, such as accumulating transcription apparatus at the key site to achieve molecular recognition showed in Figure 6. (Sabari et al., 2018). In addition, special amino acids, such as arginine, are involved in neuronal diseases that are frequently observed to be coacervated (Bhopatkar et al., 2020). Although the coacervates formed by poly R_12_ and RNA are non-toxic, poly (PR)_12_ enabled limited protein translation and exhibited cytotoxicity. Adding proline to the polyarginine structure favors multivalent interactions and governs LLPS, leading to the aggregation of acid proteins. This may offer an explanation for neuronal cell death in C9-amyotrophic lateral sclerosis.

### 5.6 Underwater adhesive

**FIGURE 7:**
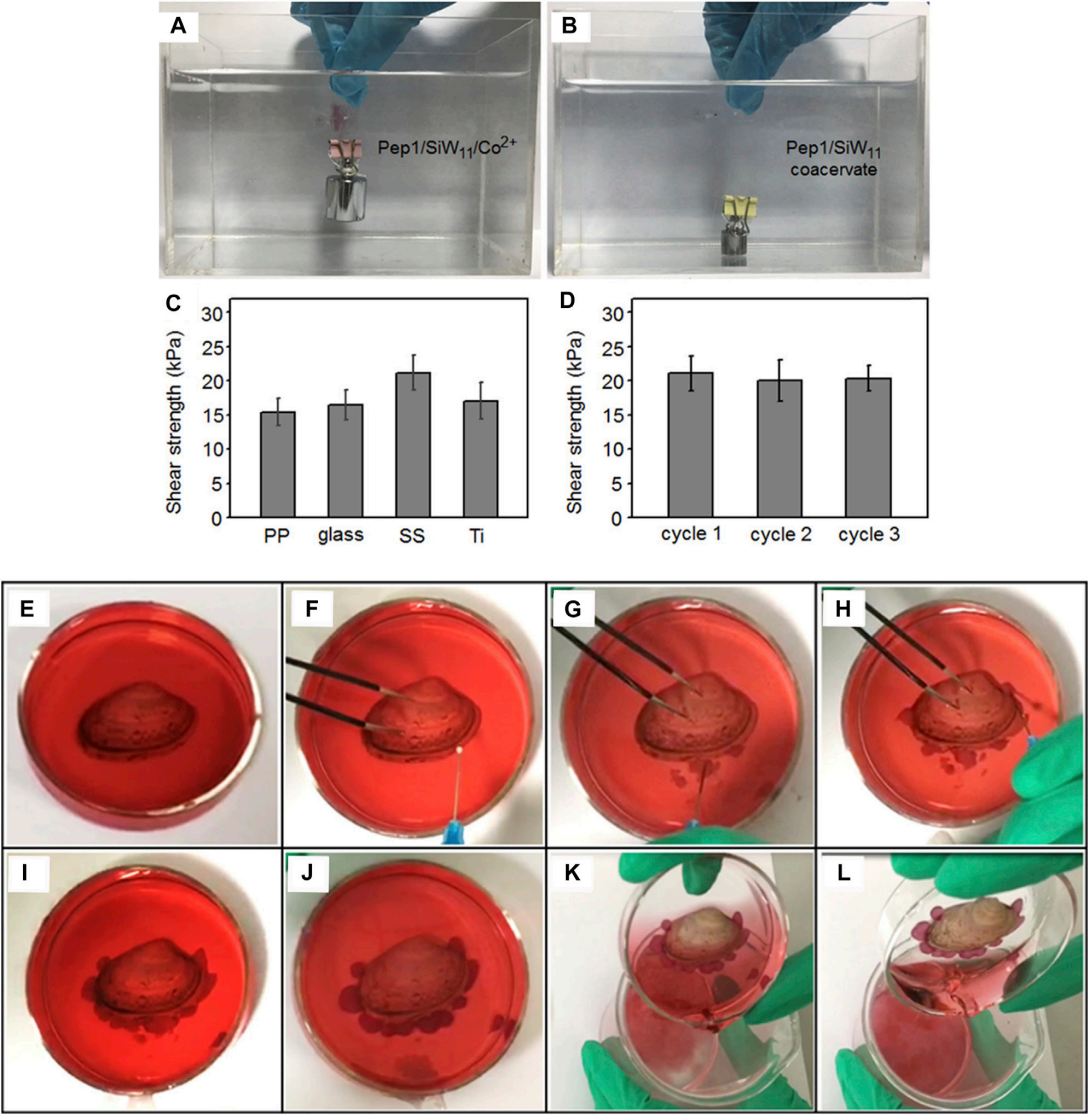
**(A)** Underwater adhesion behavior of the Pep1/SiW_11_/Co^2+^ gel (pH = 6.5) compressed between two glass slides with a 100 g load; **(B)** underwater adhesion failure of the Pep1/SiW_11_ coacervate (pH = 6.5) compressed between two glass slides with a 10 g load; **(C)** underwater shear adhesion strengths of the Pep1/SiW_11_/Co^2+^ gel bonded to different substrates (PP, glass, SS, and Ti); **(D)** underwater shear strength of the Pep1/SiW_11_/Co^2+^ gel as a function of the attachment/detachment cycles; **(E)** shell on a watch glass containing Co^2+^ aqueous solution, **(F)** injection of the Pep1/SiW_11_ coacervate through a fine needle, **(G)** coacervate droplets being injected into the Co^2+^ aqueous solution, **(H)** injected coacervate droplets adhering around the shell, **(I)** droplets after injection, **(J)** droplets changing into the gel phase after 30 min, **(K)** removing the Co^2+^ aqueous solution after the gel phase was kept for 2 h, and **(L)** tilt test demonstrating the shell to be adhered to the original site of the watch glass. (Li et al., 2019) Copyright ^©^ 2019 American Chemical Society.

Because the coacervates were easily shifted from liquid to gel by adjusting the pH value or the concentrations of the metal ions, Li el al. reported that the short peptide GHK formed droplets with anionic polyoxometalates, and the coacervation exhibited excellent mechanical stiffness and self-healing properties (Li et al., 2019). In addition, they examined the mechanical adhesion strength in different substrates, such as titanium, polypropylene, glass, and stainless steel, and the data showed that the gel had impressive adhesion strength for all four substrates. Moreover, the regulation of the pH value and metal ions could trigger the formation of peptide-based coacervates in specific locations in injectable underwater adhesives. Consequently, the coacervation produced by short peptides and polyoxometalates would be a promising method for developing underwater adhesives (Figure 7).

## 6 Conclusion and perspectives

Recent reports offer information on advances in peptide-based coacervates. Versatile peptide functional groups and secondary structures are the main driving force for liquid‒liquid phase separation and sensitivity to the cellular environment. Moreover, external stimulations influence coacervation behaviors. We summarized the currently known different types of peptide-based coacervates as well as their therapeutic applications. At present, the researches of peptide-based coacervates are mainly focused on the role of peptide contained homologous amino acid, but ignore the possibility of non-poly electrolytes peptides and their internal mechanism of dynamic process. Peptide-based coacervates participated in regulation of cell metabolism, signal transduction, gene expression, protein homeostasis and other physiological processes, and helps maintain the stability of the internal environment. Besides, the phase separation droplets are closely related to many diseases and the formation of coacervates involved in the development of therapies. Peptide-based coacervates are biocompatibility and biosafety polymers and would be beneficial to partition, protect enzymes and implement reactions in the droplet through offering substrates from eternal environment. Comprehensively, the development of peptide-based coacervates indicates that peptides have strong potential as candidates for therapeutic applications. The studies summarized in this review could shed light on the controlled structures, functions, applications of peptide-based coacervates. There are several puzzles that need to be addressed in future studies:

1) Due to the native properties of peptides that would tend to form precipitates instead of droplets, their mechanism of assembly and modulation of the methods of coacervation require further research.
2) Controllable, scalable production methods need to be developed to support the manufacture of stable and uniform coacervates.
3) Quantitative technologies are needed to evaluate the process instead of the common qualitative technology applied currently, such as fluorescence recovery after photobleaching.
4) High spatiotemporal resolution detection measurements are urgently needed to explore the changes in the molecular interface, the distribution of its components in coacervates and their mass transmission.
5) Simulation and calculations would deepen the understanding of their thermodynamics and kinetics.
6) To date, LLPS has mainly focused on biomolecules. Nanoparticles and clusters also play an important role in phase separation, and more significant applications need investigation.
